# Pathological Functions of Lysosomal Ion Channels in the Central Nervous System

**DOI:** 10.3390/ijms25126565

**Published:** 2024-06-14

**Authors:** Jianke Cen, Nan Hu, Jiawen Shen, Yongjing Gao, Huanjun Lu

**Affiliations:** Institute of Pain Medicine and Special Environmental Medicine, Co-Innovation Center of Neuroregeneration, Nantong University, Nantong 226019, China; cenjianke@outlook.com (J.C.); 17856313725@163.com (N.H.); shenjw1026@163.com (J.S.)

**Keywords:** lysosomes, ion channel, central nervous system, immune inflammation, disease

## Abstract

Lysosomes are highly dynamic organelles that maintain cellular homeostasis and regulate fundamental cellular processes by integrating multiple metabolic pathways. Lysosomal ion channels such as TRPML1-3, TPC1/2, ClC6/7, CLN7, and TMEM175 mediate the flux of Ca^2+^, Cl^−^, Na^+^, H^+^, and K^+^ across lysosomal membranes in response to osmotic stimulus, nutrient-dependent signals, and cellular stresses. These ion channels serve as the crucial transducers of cell signals and are essential for the regulation of lysosomal biogenesis, motility, membrane contact site formation, and lysosomal homeostasis. In terms of pathophysiology, genetic variations in these channel genes have been associated with the development of lysosomal storage diseases, neurodegenerative diseases, inflammation, and cancer. This review aims to discuss the current understanding of the role of these ion channels in the central nervous system and to assess their potential as drug targets.

## 1. Introduction

Intracellular organelles engage in the mutual exchange of their internal contents through various mechanisms, including vesicular and non-vesicular pathways [1]. The lysosome, an acidic organelle, serves as a degradation center and signaling hub in the cell, playing a crucial role in cellular homeostasis, development, and aging [2]. As a dynamic and heterogeneous organelle with a single limiting membrane, its morphology, size, enzyme content, and substrates vary [3]. The unique low pH characteristic of lysosomes promotes the activity of luminal hydrolytic enzymes, laying the foundation for the breakdown of macromolecules, and leading to the production of amino acids, monosaccharides, and free fatty acids [4]. Lysosomes establish membrane contact sites with the endoplasmic reticulum (ER) and mitochondria, facilitating the bidirectional transfer of metabolites and ions [5]. This interaction influences lysosomal functions, such as mobility, membrane remodeling, and repair.

Lysosomes contain over 60 soluble acidic hydrolases and 200 integral and peripheral membrane proteins [6]. The acidic hydrolases, predominantly located within the lysosomal lumen, carry out the degradation functions of the lysosome and exhibit specificity towards various substrates [7]. The lysosomal membrane proteins, encompassing a variety of transport proteins and ion channels, such as transient receptor potential mucolipin1-3 (TRPML1-3), two-pore channels 1/2 (TPC1/2), and transmembrane protein 175 (TMEM175), not only serve as a natural barrier between the acidic lysosome and the weakly basic cytoplasm, but are also necessary for lysosomal function and balanced physiological environment. These channels function in lysosomal exocytosis, membrane potential, protein degradation, autophagy, phagocytosis, and cellular signal transduction through regulating the influx and efflux of ions such as H^+^, Na^+^, K^+^, Ca^2+^, and Cl^−^ [8,9,10,11,12,13,14,15,16]. The disruption or dysfunction of lysosomal ion channels can lead to morphological abnormalities, vesicular transport defects, inflammation, and neurodegenerative diseases [17]. This review summarized the expression and functions of lysosomal channels in the central nervous system (CNS).

## 2. Lysosome Function

Lysosomes, ubiquitous organelles found in nearly all eukaryotic cells, are formed through the fusion of endosomes and vesicles budded from the trans-Golgi network [18]. Originally known as a degradation hub, lysosomes have been found responsible for metabolism, nutrient sensing, and neurologic and immune function. The diversity of lysosomal enzymes facilitates the breakdown of diverse macromolecules like nuclei acids, proteins, polysaccharides, lipids, and other substances [14]. 

Lysosomal hydrolases are synthesized in the rough ER and specifically transported through the Golgi, and then directed to lysosomes from the trans-Golgi network through a mannose-6-phosphate (M6P)-dependent transport system [19]. M6P receptors (M6PRs) aid in packaging hydrolases into clathrin-coated vesicles, delivering their contents to early endosomes, which eventually mature into late endosomes and lysosomes [20]. These enzymes are exclusively active within the acidic interior (pH 4.6–5.2) of lysosomes maintained by proton pumps. Their acid-dependent functionality prevents self-degradation in the event of lysosomal leakage or rupture, as the pH of the cell is neutral to slightly alkaline. Moreover, a continuous layer of highly glycosylated membrane proteins, mainly lysosomal-associated membrane protein (LAMP) 1 and 2, shields the luminal side of lysosomal membranes from autodigestion [15]. However, some soluble hydrolases and non-enzyme proteins are transported to lysosomes via the M6PR-independent transport, mediated by alternative receptors. For example, the trafficking of hydrolases including prosaposin and acid sphingomyelinase is mediated by neurotensin receptor 3 [21], while the transport of β-glucocerebrosidase (βGC) depends on lysosomal integral membrane protein type 2 [22]. 

Lysosomal enzymes degrade intracellular components and extracellular materials that enter the cell via endocytosis. Autophagy is classified into three types based on the mechanisms of cargo delivery: macroautophagy, chaperone-mediated autophagy, and microautophagy [23]. Macroautophagy involves the formation of autophagosomes that fuse with lysosomes, directly engulfing cytoplasmic portions into lysosomes [24], while chaperone-mediated autophagy targets specific proteins recognized by chaperones for direct delivery to lysosomes via LAMP2 receptors. Microautophagy involves the direct engulfment of cytoplasmic material by the lysosomal membrane, occurring through the invagination, protrusion, or septation of the lysosome membrane, allowing for the direct uptake of cargo. Following degradation in autolysosomes, digestion products are transported back to the cytoplasm via lysosomal membranes, facilitated by the vacuolar H^+^-ATPases (V-ATPase) and amino acid transporters [14].

Endocytic pathways, dynamin-dependent or independent, mediate the uptake of extracellular materials and membrane-bound molecules [25]. Dynamin-dependent endocytosis, including clathrin-coated pits and endophilin-mediated mechanisms, imports extracellular molecules and recycles membrane receptors [26]. Alternatively, dynamin-independent processes like macropinocytosis and phagocytosis specialize in acquiring fluid-phase molecules and ingesting large particles [27]. Once digested within lysosomes, substrates are exported into the cytoplasm through specific membrane transporters, and then utilized either as an energy source through catabolic reactions or as building blocks for anabolic reactions [28].

Lysosomes not only regulate intracellular functions but also are involved in extracellular regulation [28]. After migrating from the perinuclear region to the vicinity of the cell surface, lysosomes fuse with the plasma membrane and release their contents extracellularly [29,30]. In osteoclasts, lysosome exocytosis enables the release and activity of acidic hydrolase cathepsin K at the ruffled border and juxtaposed resorption lacuna, promoting the digestion of the bone matrix and mineral [31]. Lysosomes also play a role in the formation of extracellular vesicles, and the inhibition of the lysosome with different alkaline agents increases extracellular vesicle secretion [32].

The motility of lysosomes is not uniform; some lysosomes remain relatively stationary within the cell, while others exhibit dynamic movement along microtubules [33]. In non-polarized cells, lysosomes are positioned around the center of the microtubule organization or located at the plasma membrane and cell protrusions, while in polarized cells, they are located in the cell body, axon, and dendrites [34]. In neurons, the kinesin mediates the forward transport of lysosome to the end of the axon, and the dynein mediates the reverse transport of lysosome from the distal axon to the cell body. Dendritic microtubules exhibit mixed polarity, where both motor proteins can drive lysosomes in either direction. Lysosomes throughout axons and dendrites exhibit dynamic trafficking and positioning to meet various homeostatic demands [35]. Autophagy depends on lysosome movement [36], and the bidirectional transport of lysosomes mediates the binding of lysosomes to autophagosomes to form autolysosomes, thereby obtaining lysosomal hydrolases and an acidic environment to clear autophagosomes [37]. For example, the activity of TRPML1 has been found to promote the Ca^2+^-dependent centripetal movement of lysosomes towards the perinuclear region following autophagy induction [38].

## 3. Lysosomal Ion Channels 

**Figure 1:**
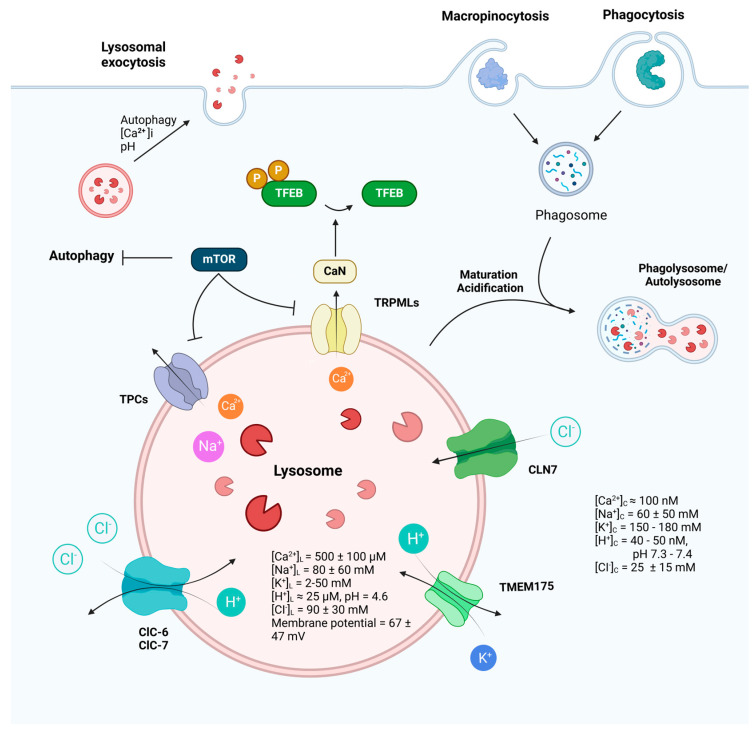
**Function and regulation of lysosomes.** Receiving inputs from both endocytic and autophagic pathways, lysosomes play a fundamental role in cellular housekeeping and metabolism, as well as critical and sophisticated roles in the immune and nervous systems. Lysosomal ion channels, by mediating ion gradients and intracellular signaling pathways, participate in various lysosomal functions, including lysosomal membrane trafficking, catabolite export, nutrient sensing, and mTOR signaling. The dynamic interplay of these ion channels not only underscores the complexity of lysosomal regulation but also highlights their indispensability in maintaining cellular health and responding to physiological challenges. (Ion concentrations in cytosol: [K^+^]_Cytosol_ = 150–180 mM [43], [Na^+^]_Cytosol_ = 10–110 mM [44,45], [Ca^2+^]_Cytosol_ = 100 nM [46], [H^+^] _Cytosol_ = 40–50 nM [47], [Cl^−^]_Cytosol_ = 25 ± 15 mM [48,49]. Ion concentrations in lysosome: [K^+^]_L_ = 2–50 mM [41,50], [Na^+^]_L_ = 80 ± 60 mM [41,50], [Ca^2+^]_L_ = 500 ± 100 μM [51], [H^+^]_L_ = 25 μM [52], [Cl^−^]_L_= 90 ± 30 mM [53,54]. Lysosomal membrane potential varies from 20 to 114 mV (luminal-side positive) [39,40,41]. The figure was created with BioRender.com.

Lysosomal ion channels are integral membrane proteins located within the lysosomal membrane, operating alongside transporters to modulate the flow of ions across the lysosomal membrane. They play a crucial role in establishing the concentration gradient for a variety of anions and cations within the lysosomes. The lumen of the lysosome is characterized by high concentrations of H^+^, Ca^2+^, and Na^+^ while exhibiting a low level of K^+^ in comparison to the cytoplasm (Figure 1). The lysosomal membrane potential is variable [39,40,41]. The concentration gradient of ions inside and outside the lysosomal membrane, as well as the open state of ion channels, determine the direction of ion movement across the cell membrane [42]. Despite their important role in the physiological and pathological processes of cells, the properties of lysosomal ion channels have remained undiscovered due to technical limitations. Recent advancements in electrophysiological techniques, especially in patch-clamp, have shed light on the identity and function of ion channels predominantly localized on lysosomes. These channels, including TRPML1-3, TPC1/2, TMEM175, and chloride channel 7 (ClC7), are activated by pH, voltage, and endogenous ligands. They exhibit varying degrees of permeability toward different ions, such as Ca^2+^, K^+^, Na^+^, H^+^, and Cl^−^, and exert a variety of functions in cargo degradation, autophagy regulation, cell signaling, and disease implication (Table 1). 

### 3.1. Ca^2+^ Channels

Calcium (Ca^2+^) stands as a pivotal second messenger, orchestrating a multitude of vital cellular processes [55]. Ca^2+^ homeostasis assumes a critical role in preserving the optimal function of lysosomes, pivotal organelles responsible for cellular waste management and recycling [56]. Channels and transporters delicately manage the influx and efflux of Ca^2+^, finely tuning essential cellular activities such as autophagy, apoptosis, and gene regulation [57]. The lysosomal Ca^2+^ concentration ([Ca^2+^]_lumen_) is around 500 μM, roughly 5000 times higher than the cytosolic concentration [51], making it the second-largest store of Ca^2+^ within the mammalian cell. However, the mechanisms of Ca^2+^ influx and maintenance of high Ca^2+^ concentration in lysosomes remain unclear. Existing researches suggests the primary source of Ca^2+^ in lysosomes is the endoplasmic reticulum [58,59]. Several studies indicated that ATP is required for Ca^2+^ transport into lysosomes in mammalian cells [60,61], and pH, temperature, heavy metals [61,62], sphingosine [63] affect Ca^2+^ transport as well. Potential lysosomal Ca^2+^ importers include Ca^2+^/H^+^ exchanger (CAX) [64], sarcoendoplasmic reticulum Ca(^2+^)-ATPase-3 pump (SERCA3) [65], and cation exchanger (solute carrier family 24 member 5) SLC24A5 [66]. Stromal interaction molecule 1 (STIM1) probably acts as a Ca^2+^ sensor in the acidic store [65]. Recently, transmembrane protein 165 (TMEM165) was identified as a novel Ca^2+^ importer in lysosomes [67], which significantly advanced understanding of lysosomal calcium entry mechanisms.

The release of Ca^2+^ from lysosomes has been extensively studied, and a plethora of ion channels are involved, such as transient receptor potential melastatin 2 [68], purinergic ATP-activated cation channel purinergic receptor (P2X4) [69], and calcium voltage-gated channel subunit alpha1 A (CACNA1a) [70]. In this review, we focus on TRPML1-3 and TPC1, 2, which are the most well-characterized.

#### 3.1.1. TRPMLs 

The TRPML family consists of three main family members in mammals: TRPML1, TRPML2, and TRPML3, sharing approximately 75% similarity in amino acid sequence [71,72,73]. These ion channels are crucial players in various cellular processes including immune inflammation [74,75,76,77]. The three members of the TRPML family are known to be activated by phosphatidylinositol 3,5-bisphosphate (PI(3,5)P2) [71,78], which is a significant component of endo-lysosomal membranes. Additionally, their activation is influenced by H^+^ concentration, which varies across different endosomal organelles and lysosomes. 

TRPML1 primarily localizes the late endolysosomal compartment in mammals [79]. Its activity peaks in the highly acidic environment of lysosomes (pH 4.6–5.2) [80,81], diminishes in weakly acidic conditions (pH 5.5), and is at its lowest under neutral conditions [82]. TRPML1 has been reported to be associated with various cellular processes including particle intake, lysosome exocytosis [83], and is also involved in phagolysosomal biogenesis, facilitating the fusion of phagosomes and lysosomes, ensuring proper cellular disposal [84]. Moreover, TRPML1 participates in autophagy by interacting with mTOR pathway. During starvation, reduced mTOR levels activate TRPML1, which subsequently promotes mTOR activity necessary for autophagic lysosome reformation [85]. 

TRPML2 exhibits a predominant localization within the long tubular recycling endosome, lysosomes, and plasma membrane [86]. It functions as an inwardly rectifying ion channel that allows calcium ions to permeate and is most active at pH 7.2 [87]. It is primarily regulated by extracytosolic protons, rather than Ca^2+^ levels [82], or linoleic acid, which is known to activate other transient receptor potential (TRP) [88]. Although it was not directly associated with any disease phenotype, TRPML2 is involved in various cellular processes, including endocytosis, membrane trafficking, and lysosomal function [86]. Recent studies have discovered its new role in host defense against various pathogens, including bacteria, viruses, and fungi. Activation of toll-like receptors (TLRs) such as TLR2, TLR3, TLR4, TLR7, and TLR8 leads to a significant increase in TRPML2 expression [76]. Additionally, TRPML2 activation directly stimulates the secretion of the chemokine CCL2 by bone marrow-derived macrophages, indicating its involvement in chemokine transport and secretion in mouse macrophages [89].

Overexpressed TRPML3 is observed at various cellular locations, including the plasma membrane (PM), ER, autophagosomes, lysosomes, early and late-endosomes [90]. TRPML3 can be activated by PI(3,5)P2 and Phosphatidylinositol-3-phosphate (PI3P) [91,92]. Meanwhile, high luminal pH (pH 6–6.5) and substitution of luminal Na^+^ with K^+^ augment TRPML3 activity [93]. It is found within all autophagosomes, including those present in both feeding and stressed cells, and is recruited into newly formed autophagosomes in stressed cells [94], suggesting its important role in autophagy. Unlike TRPML1, TRPML3 appears to inhibit mTOR through a positive feedback mechanism. Xu et al. recently found that under nutrient starvation, reduction in mTOR stimulates TRPML3 through recruitment of TRPML3 from endolysosome onto phagophores where pH and PI3P levels are higher. Ca^2+^ released via TRPML3 then activates large conductance calcium-activated potassium channels (BK), which in turn facilitates further Ca^2+^ release through TRPML3, potentially by removing luminal Na^+^ inhibition. In addition, TRPML3/BK complex facilitates mTOR inhibition and autophagy induction [93]. TRPML3 also plays a role in immunity. With elevated expression of TRPML3 in lung tissue, primarily concentrated within alveolar macrophages (AMΦ). These cells, integral to the immune system, are responsible for clearing harmful particles and microorganisms from the respiratory tract [95].

#### 3.1.2. TPC Channels

TPCs are ligand-gated cation-selective ion channels, located in the membranes of acidic organelles, such as endosomes, lysosomes, and endolysosome [96]. Characterized by their unique structure that includes two pore-forming domains per subunit, TPCs are capable of conducting ions, notably Ca^2+^ and Na^+^, in response to changes in membrane potential and intracellular signaling molecules [97]. 

The TPC family consists of three members, each exhibiting distinct localization and biophysical characteristics. TPC1 and TPC2 are localized in endosomes and lysosomes, while TPC3 is present in plasma membranes [98]. TPC1 and TPC2 act as Ca^2+^ release channels in response to stimuli such as nicotinic acid adenine dinucleotide phosphate (NAADP) [99]. However, when activated by phosphatidylinositol 4,5-bisphosphate (PI(4,5)P2), they release Na^+^ instead of Ca^2+^. In addition, Ca^2+^, pH, ATP, and voltage can regulate the activities of TPC1 and TPC2 [100]. They contribute to processes such as phagocytosis, endocytosis, and lysosomal exocytosis by modulating fusion and fission events within endolysosomal compartments [101]. Dysregulation of TPC activity has been implicated in various physiological and pathological conditions. For example, levels of TPC1 and TPC2 are elevated in left ventricular samples obtained from both ischemic and dilated hearts of patients with heart failure [102], absence of TPC2 in hepatocytes leads to various phenotypes consistent with non-alcoholic fatty liver disease (NAFLD) [103]; knockdown or knockout of either *Tpc1* or *Tpc2* prevents Ebola virus infection in vitro [104].

In addition, TPCs are widely expressed across various cells in the immune system such as B cells, T cells, dendritic cells, NK cells, and mast cells. Notably, macrophages exhibit a particularly high level of expression of both TPC1 and TPC2, indicating their significant role in the function of these immune cells [105]. As mentioned, TPCs are integral components of this signaling cascade activated by NAADP, which selectively stimulates exocytosis crucial to immune cell function, particularly in cytotoxic T lymphocytes (CTLs) during immune synapse formation [106]. TPCs translocate to immune synapses upon CTL activation, facilitating Ca^2+^-dependent exocytosis of cytolytic particles for cell killing [107].

Furthermore, TPCs have been implicated in regulating calcium homeostasis within endolysosomal compartments, influencing mast cell reactivity [96]. Mast cells are vital components of the innate immune system and play pivotal roles in defending against pathogens and orchestrating allergic responses. Dysregulation of TPC-mediated calcium signaling can lead to excessive release of histamine and heparin, contributing to allergic reactions [106]. Studies have shown that TPC1 regulates calcium homeostasis, modulating the feedforward cycle involving inositol triphosphate receptors and leading to increased exocytosis and allergic reactions when inhibited [106,107].

### 3.2. Cl^−^ Channels

As the most abundant physiological anion, chloride (Cl^−^) is widely distributed in cytoplasm and vesicule/organel [108]. Its transport is involved in numerous physiological processes including regulation of cell excitability, transepithelial material transport, cell volume regulation, and organelle acidification [108,109,110]. The chloride channel (ClC) and ceroid lipofuscinosis neuronal (CLN) family contribute to regulation of Cl^−^ in lysosome.

#### 3.2.1. ClC Family

The ClC family has been extensively studied, mainly acting as Cl^−^ channels or 2Cl^−^/H^+^ exchangers. There are nine members present in mammals (ClC1 to ClC7, ClCKa, and ClCKb). Four of these proteins, ClC1, ClC2, ClCKa, and ClCKb, serve as Cl^−^ channels, essential for stabilizing membrane potential and ion homeostasis across the plasma membrane [108]. Other ClC proteins are primarily expressed in intracellular organelles like endosomes and lysosomes, where they are probably important for proper luminal acidification, in synergy with the V-ATPase [111]. ClC3 to ClC5 function as endosomal 2Cl^−^/H^+^ exchanger [112], while ClC6 and ClC7 are late endosomal/lysosomal 2Cl^−^/H^+^ exchanger [113,114,115,116]. The sorting of endo-lysosomal transmembrane proteins is generally mediated by cytosolic motifs that are recognized by adaptor proteins. For instance, a basic amino acid stretch is crucial for the localization of heterologously expressed ClC-6 to early and/or recycling endosomes [117], and the N terminus of ClC-7 contains binding motifs for both AP and GGA adaptors, which are important for its lysosomal targeting [118]. ClC6 and ClC7 share significant sequence homology and structural similarities [119]. Patients with mutation in *ClCn6* (ClC6 encoding gene) exhibit neurodegeneration and West Syndrome [120,121], while mice lacking *ClCn6* only develop a mild neuronal lysosomal storage disorder, primarily affecting the initial axon segment, but do not display any discernible phenotype [115]. *ClCn7* (ClC7 encoding gene) has been identified as the causal gene in a distinctive phenotype encompassing osteopetrosis [122], renal tubule acidosis [123], and blindness [124].

#### 3.2.2. CLNs 

CLN proteins are distributed throughout the endomembrane system and regulate a variety of cellular processes related to ion homeostasis, autophagy, lipid metabolism, and protein trafficking [125]. To date, fourteen CLN proteins (CLN1-14) have been identified and characterized [126,127]. Among these, CLN6 and CLN8 localize to the membrane of the ER, where they regulate lysosomal enzyme recruitment and lysosome biogenesis [128], while CLN1, CLN2, CLN3, CLN5, and CLN10 localize to lysosomes and function as soluble enzymes [129,130,131]. 

In recent years, CLN7 has been recognized as a novel endolysosomal Cl^−^ channel [132]. Research indicates that overexpression of CLN7 enhances endolysosomal chloride currents and promotes the enlargement of endolysosomes through a Ca^2+^/calmodulin-dependent mechanism [132]. CLN7 also regulates lysosomal chloride conductance, luminal pH, and lysosomal membrane potential, promoting the release of lysosomal Ca^2+^ through TRPML1 [132].

### 3.3. K^+^ and H^+^ Channels 

TMEM175, also known as the transmembrane protein 175 channel, serves as the principal mediator of K^+^ and H^+^ permeability within lysosomes and late endosomes [133,134]. It is a growth-factor-activated and AKT (protein kinase B)-gated lysosomal ion channel [135], which comprises two sets of repeating 6-transmembrane segments organized in an hourglass-like configuration [133]. The absence of TMEM175 in lysosomes results in the loss of K^+^ conductance, insensitivity to K^+^ currents, and disruption of lumen pH stability, thereby leading to abnormal fusion events during autophagy [133,136]. Compounds such as arachidonic acid and small synthetic chemicals like DCPIB and ML 67-33 have been identified as activators of TMEM175-mediated H^+^ and K^+^ currents [134]. Loss of TMEM175 triggers the rapid buildup of α-synuclein within lysosomes due to accelerated fusion between autophagosomes and lysosomes, ultimately culminating in neuronal cell demise. TMEM175 plays a particularly important role in regulating lysosomal pH homeostasis. When the lysosomal lumen experiences hyperacidification induced by the V-ATPase, TMEM175 is activated and precipitates a rapid surge in H^+^ efflux [134]. Overexpression of TMEM175 promotes lysosomal alkalization, while its deletion exacerbates lysosomal acidification and diminishes the activity of essential hydrolases, including aspartase, cysteine protease, βGC, cathepsin D, and cathepsin B, resulting in impaired lysosomal autophagy [137,138,139,140].

## 4. The Role of Lysosomal Ion Channels in the Nervous System

Lysosomes play a pivotal role within the CNS, contributing to various essential functions through degradation and signaling mechanisms. In recent years, lysosomes have garnered significant attention in the study of the late-onset neurodegenerative diseases [141]. The proper operation of lysosomal ion channels is crucial not only for synaptic transmission and plasticity but also holds significance in the pathogenesis and potential treatment of neurodegenerative diseases. 

### 4.1. Physiological Functions of Lysosomal Ion Channels in the Nervous System

TRPML3, identified in sensory neurons, cochlea, and melanocytes, gained prominence due to its association with the Varitint-waddler (Va) mouse phenotype, characterized by hearing loss, circling behaviour, pigmentation defects, and embryonic lethality [142]. In hair cells, TRPML3 is critical for stereocilia bundle formation during development and may function during endocytosis or exocytosis [142]. Mutations in TRPML3 have been identified as the cause of cochlear abnormalities, including disorganization and fusion of stereocilia, distortions in both the apical and distal regions of inner and outer hair cells, as well as loss of pigmented intermediate cells in the stria vascularis, leading to hearing impairment [142]. Notably, conditionally inactivating *Trpml3* in mice did not lead to circling behavior, balance impairment, or hearing loss observed in the Va mouse model [143]. In the dorsal root ganglion, TRPML3 shows a very low expression, but exhibits a significant increase in mRNA levels following spared nerve injury. Widespread expression of TRPML3 is observed in nociceptors and mechanoreceptors after spared nerve injury, indicating its potential role in neuropathic pain [144]. 

In cortical neurons, TRPML1 and TPC2 transcripts are detectable, while the endolysosomal cation channels including TRPML2, TRPML3, and TPC1 were largely undetectable [145]. TRPML1 activation promotes both inward and outward lysosomal trafficking in dendrites through dynein-dependent mechanisms [146]. When induced by disinhibition from LAMTOR1 or pharmacological enhancement, activated TRPML1 enhances lysosomal trafficking, resulting in changes in synaptic plasticity, which favors long-term depression (LTD) induction in the adult hippocampal CA1 region, while impeding long-term potentiation (LTP) consolidation and affecting learning and memory process [146]. TPCs are implicated as central figures in neuronal excitatory signaling driven by NAADP [147]. Elevated [Ca^2+^] triggers calcium-induced calcium release, promoting critical processes such as LTP and potentially contributing to the pathogenesis of neurodegenerative diseases [148]. Additionally, TPCs may play a pivotal role in glutamate-induced autophagy of glial cells, although there is conflicting evidence regarding their involvement in lysosomal stabilization and the regulation of autophagic flux [149]. Recent studies have shown that TPC2-mediated calcium signaling promotes axon growth in zebrafish’s caudal primary motor neurons [150]. This association was established through the application of the NAADP receptor antagonist trans-Ned 19, along with genetic silencing techniques [150].

TPC2 is implicated in social interaction. In hypothalamic neurons, TPC provides critical Ca^2+^ signals to increase the releasable large dense-cored vesicles for exocytosis, which stores oxytocin, a prominent regulator of many aspects of mammalian social behaviors [151]. Knockout or inhibition of TPC in mice leads to a significant reduction in plasma oxytocin levels, impaired oxytocin secretion, and social defects, highlighting the importance of neuropeptide vesicle priming for activity-dependent release [151]. Furthermore, activation of type 1 metabotropic glutamate receptors sustains somatodendritic oxytocin release by recruiting TPCs [151].

Overall, lysosomal ion channels play diverse and essential roles in the nervous system, impacting various physiological processes and contributing to the pathogenesis of neurodegenerative diseases.

### 4.2. Role of Lysosomal Ion Channel in Nervous Disease

#### 4.2.1. Lysosomal Storage Disorders (LSDs)

The LSDs are inherited metabolic disorders characterized by abnormal accumulation of macromolecular substrates due to lysosomal dysfunction [152]. Neurological symptoms or signs are prevalent in most LSDs, including developmental delays, seizures, acroparesthesia, motor weakness, premature mortality, and extrapyramidal manifestations [153]. Emerging evidence suggests that ion channels in the endolysosomal system play a crucial role in the pathology of neurodegenerative LSDs [154]. 

LSDs can be categorized based on the type of accumulated storage material, including sphingolipidoses, mucopolysaccharidoses, mucolipidoses (ML), and neuronal ceroid lipofuscinoses (NCL) [155]. Within the ML subgroup, there are four distinct types: MLI, MLII, MLIII, and MLIV. MLIV is a debilitating disease marked by infantile-onset visual impairment, motor dysfunction, and neurodegeneration, constituting the most direct link between defective lysosomal Ca^2+^ release and neurodegeneration, attributed to dysfunction of the lysosomal cation channel TRPML1 [156].

TPC2, sharing several features with TRPML1, is also implicated in LSDs. Both channels are permeable for Ca^2+^ and Na^+^, reside in endolysosomal membranes, are activated by PI(3,5)P2, cause trafficking defects when lost, interact with mTOR/Transcription factor EB (TFEB)/autophagy pathways, and promote lysosomal exocytosis [145]. TPC2 is widely expressed in the CNS, with both neurons and glia expressing TPC2 in various brain regions, including the corpus callosum, hippocampus, and cerebellum [145]. Intravenous administration of a TPC2 agonist reduces the accumulation of storage materials in the CNS and restores motor performance in mouse model of the MLIV [145,157].

NCLs also known as batten disease, are characterized by seizures and visual, cognitive decline, and motor deficits, culminating in premature death [158]. The important roles of ClC6 and ClC7 in NCL have been revealed, with their influence on Cl^−^accumulation which consequently affects protein degradation [41,159,160,161,162,163]. Loss of *Clc6* lead to the mild neuropathologic features, such as the accumulation of autofluorescent [164], while NCL phenotypes caused by ClC7 are more typical including lysosomal storage, retinal and optic nerve degeneration, and selective loss of hippocampal and cerebellar neurons [159,164]. Addtionally, *CLCN7* variant is associated with developmental delay and hypopigmentation due to lysosomal swelling, abnormal storage and hyperacidity [165,166]. The participation of V-ATPase in the lysosomal acidification process is also noteworthy in NCL. Dysfunction of V-ATPase is observed in *Cln1*^−/−^ mice, an infantile NCL model [167]. Furthermore, TFEB-V-ATPase axis is involved in lysosomal biogenesis [168] and regulation of microglia and immune response in tauopathy and Alzheimer’s disease (AD) [169]. Besides, each *CLN* gene is associated with a specific subtype of the disease, varying in severity and age of onset [127,158]. For instance, homozygous frameshift mutation in the *CLN7* gene of macaque recapitulates key features of human NCLs, including behavioral and neuropathological deficits such as visual impairment, tremor, incoordination, ataxia, and impaired balance [170]. In *Cln7*-deficient mouse embryonic fibroblasts (MEFs), CLN5 protein levels are significantly reduced, suggesting a potential role of CLN5 in the pathogenesis of CLN7 disease [171]. TFEB activation by tamoxifen reduces lysosomal accumulation of storage material in CLN7 type NCL patient-derived induced pluripotent stem cells (iPSC), indicating a therapeutic potential for TFEB modulation [172].

#### 4.2.2. Niemann Pick Disease (NPD)

NPD is a rare congenital recessive disorder characterized by lipid accumulation in various organs [173], leading to a range of clinical manifestations. Patients may exhibit lipid storage and foam cell infiltration in tissues, along with overlapping features including hepatosplenomegaly, pulmonary insufficiency, and/or CNS involvement. NPD has four subgroups (A, B, C1, C2) distinguished by age of onset, clinical presentation, and sphingomyelin storage patterns [174]. 

Types A and B result from mutations in the *SMPD1* gene [175]. Type A NPD patients typically manifest within the first year of life with hepatosplenomegaly and failure to thrive. It follows a rapidly progressive neurodegenerative course, marked by profound hypotonia and developmental delays, often resulting in early mortality [176]. In contrast, NPC is characterized by mutations in the *NPC1* gene (95% of cases), with a minority attributable to *NPC2* gene (4%), and potentially other genes unidentified genes (1%) [177]. NPC patients do not exhibit severe sphingomyelinase deficiency, but present with sub-acute nervous system involvement and moderate visceral storage pathology [178]. While NPC1 and NPC2 proteins are essential for proper transport and metabolism of cholesterol and other lipids within cells [179], accumulation of cholesterol is observed in about 80% of the NPC cases [178]. 

Neurological symptoms of NPC commonly include cerebellar ataxia, dysarthria, dysphagia, progressive dementia, and occasionally seizures [178]. Recent research has shown promising approaches for the treatment, including pharmacological and genetic activation of TRPML1 to ameliorate lipid trafficking defects and cholesterol accumulation associated with NPC1 [180]. Additionally, activation of TPC2 using the small molecule agonist has demonstrated efficacy in reducing cholesterol accumulation in patient fibroblasts while promoting lysosomal exocytosis and autophagy, processes often impaired in NPC1 [145].

#### 4.2.3. Neurodegenerative Disease 

The dysfunction of these ion channels can lead to impaired lysosomal function, which is increasingly recognized as a significant contributor to the pathology of neurodegenerative diseases. Modulating the activity of these channels holds promise for restoring proper lysosomal function, potentially slowing or even halting the progression of these debilitating diseases.

(a) Alzheimer’s disease (AD)

AD is the predominant form of dementia, accounting for 60–70% of all diagnosed cases of cognitive decline [181]. AD manifests with symptoms including memory loss, repetitive behavior, misplacing items, and difficulties in daily activities. Genetic mutations in genes such as *APP, PSEN1, PSEN2*, and *APOE* can contribute to both early and late-onset forms of AD [182,183]. Key hallmarks of AD pathology include the accumulation of amyloid-beta plaques and hyperphosphorylated tau protein in neurofibrillary tangles. 

The relationship between lysosomes and AD is complex and involves multiple mechanisms. Various AD risk genes, including *BIN1, CD2AP, PICALM, PLD3, SORL1,* and *TREM2,* are linked to the endosomal-autophagic-lysosomal (EAL) system. Early studies have suggested that enhancing lysosomal function could potentially mitigate key AD hallmarks, such as tau accumulation [184]. Recent research investigates the role of TRPML1 in EAL dysfunction associated with AD. Abnormal TRPML1-mediated Ca^2+^ release and endolysosomal abnormalities have been observed in AD brains and neurons expressing APOE ε4-, a major AD risk factor. Furthermore, inhibiting phosphoinositide kinase FYVE (PIKfyve) induces AD-like EAL defects in neurons, which can be rescued by TRPML1 agonist ML-SA1 [185]. Additionally, activation of TRPML1 has been shown to facilitate the expulsion of accumulated tau through lysosomal exocytosis [186] and eliminate amyloid-β from lysosomal compartments in neurons [187]. These findings highlight TRPML1 as a promising therapeutic target for AD.

(b) Parkinson’s disease (PD)

PD is a prevalent neurodegenerative disorder characterized by symptoms such as tremors, bradykinesia (slowed movement), muscle stiffness, impaired balance, and cognitive impairment [188]. It can be caused by genetic mutations in various genes, including *SCNA, LRRK2, PINK1, and PLA2G6* [189], or by exposure to certain chemicals such as phenothiazines [190], polychlorinated biphenyls (PCBs) [190], and organochlorines [191]. Mitochondrial dysfunction, oxidative stress, and the accumulation of α-synuclein protein contribute to the formation of Lewy bodies and progressive degeneration of the dopaminergic system in the brain [192]. The lysosome, responsible for protein degradation and recycling, plays a crucial role in PD pathology [193].

Studies using fibroblasts from PD patients have revealed an expansion of the lysosomal compartment in these cells, a phenomenon mitigated by treatment with TPC/NAADP antagonists Ned19 or genetic knockdown of *Tpc2* [194]. Moreover, overexpression of dominant-negative TPC2 or NAADP antagonism hindered autophagic induction mediated by the PD-associated LRRK2 protein [195]. However, further investigations are needed to elucidate the role of TPC2 in neural cells, particularly in iPSC-derived human neurons, and its impact on PD-associated α-synuclein aggregation and Lewy body formation [196]. Additionally, exploring the effects of different mechanisms of TPC2 activation, such as by PI(3,5)P2 or NAADP, on lysosomal function, autophagy, and cell viability in PD and lysosomal storage disorders is essential. While TPC2 antagonists could restore lysosomal Ca^2+^ and pH and rescue autophagy [197], prolonged inhibition of TPC2 may result in trafficking defects and cholesterol overload, posing potential risks for neurodegenerative disease development [103]. Therefore, understanding the complex role of TPC2 in lysosomal function and neurodegeneration is critical for developing targeted therapeutic interventions for PD and related disorders.

Earlier genome-wide association studies have uncovered the potential significance of genetic variations in TMEM175 concerning PD [198]. Recent research has demonstrated that elevated expression or activity of TMEM175 may suppress autophagy and promote apoptosis, potentially leading to neuronal demise in PD. Conversely, knockout of *Tmem175* has shown significant neuroprotection in a mouse model of PD induced by MPTP without causing notable adverse effects [199]. However, contrasting findings indicate that deficiency in TMEM175 results in the loss of dopaminergic neurons and impairs motor function in mice. Additionally, a TMEM175 loss-of-function variant has been nominally associated with accelerated rates of cognitive and motor decline in humans with PD [135].

(c) Amyotrophic lateral sclerosis (ALS)

ALS is a rare neurodegenerative disease of the motor neurons, characterized by progressive voluntary muscle weakness [200]. Onset and development of ALS are associated with lysosome/autophagy pathway. TRPML1 agonist ML-SA1 induces lysosomal Ca^2+^ in motor neuronal cells and protects motor neurons from the neurotoxicity of cyanobacterial toxin beta-methylamino-L-alanine by promoting autophagic clearance and counteracting ER stress [201], which accumulates in CNS of the ALS/Parkinson-dementia complex patients [202]. Besides, genetic studies identify mutations in the factor-induced gene 4 (FIG4) as a cause of familial ALS [203]. FIG4 regulates cellular levels of PI(3,5)P2 [204], with its deficiency impairing biosynthesis of PI(3,5)P2, the endogenous ligand of endolysosomal TRPML, and two-pore channels. It is worth to note that mutations in the *FIG4* gene present a comparatively rare etiological factor in ALS pathology, particularly compared to other contributors related to lysosome and autophagy function, such as gene *C9orf72* and *FUS* [205,206].

#### 4.2.4. Stroke

Stroke, a prevalent cerebrovascular condition worldwide, is characterized by post-ischemic reperfusion, which stands out as a significant contributor to delayed secondary brain injury [207]. In the oxygen-glucose deprivation/reoxygenation (OGD/R) model, a reduction in TMEM175 levels was observed in neurons, while upregulation of TMEM175 demonstrated a dual effect by mitigating mitochondrial inactivation and protecting neurons from ischemic injury. Moreover, TMEM175 restoration facilitated lysosomal internal environment homeostasis and reinstated hydrolase activity. Consequently, heightened cathepsin D activity ensued, rendering neurons resistant to OGD-induced damage [139]. While the ramifications of TMEM175 deficiency in ischemia-reperfusion injury remain unknown, it is plausible to speculate its crucial involvement in this pathological process.

During ischemic reperfusion, the autophagic pathways in various brain cells are activated [208], leading to neuronal necrosis or apoptosis. Considering that ischemia results in hypoxia and excessive glutamate release, excessive activation of autophagic flux occurs [209]. In a transient focal ischemia model, pharmacological inhibition of the TPC2 can rescue primary cortical neurons from hypoxia-induced death, interfere with autophagy, impede excessive autophagic flux, and reduce the infarct area in rats with focal ischemia [210]. Post-ischemic hypoxic neuronal protein homeostasis dysfunction, endoplasmic reticulum stress, and glutamate receptor-mediated excitotoxicity may be improved following TPC2 inhibition. Moreover, the TPC2 lysosomal channel is likely a putative target in this cascade reaction [210]. 

## 5. Conclusions and Future Perspectives

Lysosomes regulate a broad spectrum of vital cellular functions, including cellular clearance, autophagy, recycling and gene expression [17]. Within lysosomes, ion channels fulfill distinct functions (Figure 1). For instance, Ca^2+^ channels, play a crucial role in regulating processes such as autophagy, membrane fusion and division. Meanwhile, Na^+^ and K^+^ channels primarily modulate the membrane potential of lysosomes, thereby influencing their activity and function. Additionally, H^+^ channels are primarily involved in regulating the pH value of lysosomes, ensuring optimal conditions for lysosomal enzyme activity and cellular degradation processes. Furthermore, interplay between lysosomal ion channels and transporters has been illuminated based on mathematical models: ClC7 function as 2Cl^−^/H^+^ exchanger, interestingly, its disruption impinge on luminal concentrations of Ca^2+^, Na^+^, K^+^ [211], affecting acidification. A counterion flux, involving anions (like Cl^−^ entering the lumen) and/or cations (like K^+^ and Na^+^ leaving the lumen), is necessary to neutralize the membrane potential generated by the V-ATPase [212].

Lysosomal ion channels play multifaceted roles in the CNS, as well as in the immune systems, exerting significant influence on various physiological and pathological processes. Through their regulation of ion homeostasis, pH balance, and vesicular trafficking [101,213], these channels contribute to fundamental cellular functions such as autophagy [214] and immune response modulation [105]. Dysregulation of these channels has been implicated in a myriad of neurological and immunological disorders, emphasizing their potential as therapeutic targets for intervention. 

Certain diseases directly result from mutations or variations in genes encoding lysosomal ion channels, correcting the function of these channels is a logical and promising approach to treatment. For example, mutations in *Trpml1* can result in MLIV [191], a severe lysosomal storage disorder characterized by developmental delays, intellectual disability, and impaired vision and motor function. Although existing studies have utilized compounds such as small molecules, peptides, micro-RNAs, and antibodies to target plasma membrane ion channels, organelle channels, particularly lysosomal ion channels, remain largely unexplored as targets for drug development [215]. However, promising research has shown that synthetic TRPML1 agonist ML-SA1 can repair muscle cell damage, alleviate abnormal lysosomal storage [216], and rescue AD-like EAL pathogenesis [185]. An enzyme replacement therapy represents the first treatment to delay the progression of CLN2 NCL, it involves the administration of the enzyme, a recombinant lysosomal enzyme named cerliponase alfa, directly into the lateral cerebral ventricles to target the central nervous system [217]. Concurrently with this significant milestone, numerous research groups are employing diverse therapeutic approaches to expedite the creation of innovative treatments for different types of NCL at an unprecedented speed.

A deeper understanding of the physiological roles of lysosomal ion channels could unveil novel therapeutic strategies for conditions characterized by dysregulated immune responses and neuroinflammation [218]. Considering the distinct physiological functions and substantial potential for drug development associated with lysosomal ion channels, future research in this realm is poised to advance, paving the way for breakthroughs not only in the field of ion channels but also in broader areas of cellular physiology, neurobiology, and therapeutic interventions.

## Figures and Tables

**Table 1:** Major lysosomal ion channels and their functions.

	Permeability	Subcellular Location	Optimal pH	Agonists	Inhibitors	Functions	Phenotype
TRPML1	Ca^2+^, Fe^2+^, Zn^2+^, Na^+^, K^+^, Cs^+^, and Mn^2+^	LE and LY	4.6–5.2	ML-SA1, SF-51, and PI(3,5)P2	PI(4,5)P2, and ML-SI	Lysosomal endocytosis, phagolysosomal biogenesis, membrane transport, and autophagy	ML-IV, NPC, AD, and hyperdistended/hypertrophic bladder
TRPML2	Ca^2+^, Fe^2+,^ and Na^+^	EE, RE, LE, LY, and PM	7.2	ML2-SAI, PI(3,5)P2, SF-21, SF-41, and SF-81	ML-SI	Membrane transport, ARF6-regulated recirculation pathways, macrophage migration, and CCL2 secretion	
TRPML3	Ca^2+^ and K^+^	EE, LE, LY, and PM	6–6.5	ML-SA1, SF-51, PI(3,5)P2, PI3P, SF-21, SF-41, and SF-81	PI(4,5)P2 and ML-SI	Membrane transport and autophagy	Deafness and circling behavior, Va, iron-deficient anemia, emphysema, and chronic obstructive pulmonary
TPC1	Ca^2+^, K^+^, Li^+^, and Na^+^	EE and LY	↑	PI(3,5)P2, and NAADP	Ned-19	Acrosome reaction, lysosome membrane potential, pH stability, amino acid homeostasis, and mTOR-dependent nutrient sensing	MLIV
TPC2	Na^+^ and Ca^2+^	LE and LY	↑	PI(3,5)P2, TPC2-A1-P, and NAADP	Ned-19	Autophagy and mTOR-dependent nutrient sensing	MLIV, virus trafficking, and NPC1
ClC6	H^+^ and Cl^−^	LE, LY, and ER	↓			pH stability	NCL
ClC7	H^+^ and Cl^−^	LE and LY	↑			pH stability	NCL, osteopetrosis, renal tubule acidosis, epilepsy, and blindness
TMEM175	K^+^ and H^+^	EE, LE, and LY	↑	AKT and Growth factor	4-AP and Zn^2+^	Autophagy, pH stability, dynamics of α-synuclein, energy production, and apoptosis	PD, dementia, and multisystem atrophy
CLN7	Cl^−^	LE and LY	↓		DIDS, NFA, and NPPB	Lysosomal membrane potential and lysosomal fusion	NCL and macular dystrophy

## Data Availability

Not applicable.

## Abbreviations

AD, Alzheimer’s Disease; AMΦ, Alveolar Macrophages; BMDM, Bone Marrow-Derived Macrophages; ClC, Chloride Channel; CLN, Ceroid Lipofuscinosis Neuronal; CNS, Central Nervous System; CMA, Chaperone-Mediated Autophagy; CTLs, Cytotoxic T Lymphocytes; EAL, Eal Domain; EE, Early endosome; ER, Endoplasmic Reticulum; FIG4, Factor-induced Gene 4; iPSC, induced Pluripotent Stem Cells; LacCer, Lactosylceramide; LAMP, Lysosomal-associated membrane protein; LE, Late endosome; LY, Lysosome; LSDs, lysosomal storage disorders; LTD, Long-Term Depression; LTP, Long-Term Potentiation; M6P, Mannose 6-Phosphate; M6PR, Mannose-6-phosphate receptor; MEFs, Mouse Embryonic Fibroblasts; ML, Mucolipidosis; NAADP, Nicotinic Acid Adenine Dinucleotide Phosphate; NAFLD, Non-Alcoholic Fatty Liver Disease; NCL, Neuronal Ceroid Lipofuscinoses; NPA, Niemann-Pick disease type A; NPC, Niemann-Pick disease type C; OGD/R, Oxygen-Glucose Deprivation/Reperfusion; PD, Parkinson’s Disease; PI(3,5)P2, Phosphatidylinositol 3,5-bisphosphate; PI(4,5)P2, Phosphatidylinositol 4,5-bisphosphate; PI3P, Phosphatidylinositol-3-phosphate; PM, Plasma Membrane; PCBs, Polychlorinated Biphenyls; PIKfyve, Phosphoinositide Kinase, FYVE-type Zinc Finger Containing; RE, Recycling endosome; SNI, Spared Nerve Injury; TFEB, Transcription Factor EB; TPC, Two-Pore Channel; TMEM175, Transmembrane Protein 175; TRPML, Transient Receptor Potential Mucolipin; Va, Varitint-waddler; V-ATPase, vacuolar H+-ATPases; βGC, β-glucocerebrosidase.
